# Epidemiological characteristics of traumatic spinal cord injuries in a multicenter retrospective study in northwest China, 2017–2020

**DOI:** 10.3389/fsurg.2022.994536

**Published:** 2022-10-10

**Authors:** Xiaohui Wang, Jinpeng Du, Chao Jiang, Yong-yuan Zhang, Fang Tian, Zhe Chen, Yuyang Zhang, Ying Zhang, Liang Yan, Dingjun Hao

**Affiliations:** ^1^ Department of Spinal Surgery, Honghui Hospital, Xi’an Jiaotong University, Xi'an, China; ^2^ Department of Orthopaedic, The First Affiliated Hospital of Xi’an Jiaotong University, Xi'an, China; ^3^ Orthopaedic Third Ward, Yulin No. 2 Hospital, Yulin, China

**Keywords:** trauma, epidemiology, traumatic spinal cord injuries, COVID-19, northwest China

## Abstract

**Background:**

Traumatic spinal cord injuries (TSCIs) are worldwide public health problems that are difficult to cure and impose a substantial economic burden on society. There has been a lack of extensive multicenter review of TSCI epidemiology in northwest China during the Corona Virus Disease 2019 (COVID-19) pandemic.

**Method:**

A multicenter retrospective study of 14 selected hospitals in two provinces in northwest China was conducted on patients admitted for TSCI between 2017 and 2020. Variables assessed included patient demographics, etiology, segmental distribution, treatment, waiting time for treatment, and outcomes.

**Results:**

The number of patients with TSCI showed an increasing trend from 2017 to 2019, while there were 12.8% fewer patients in 2020 than in 2019. The male-to-female ratio was 3.67:1, and the mean age was 48 ± 14.9 years. The primary cause of TSCI was high falls (38.8%), slip falls/low falls (27.7%), traffic accidents (23.9%), sports (2.6%), and other factors (7.0%). The segmental distribution showed a bimodal pattern, peak segments were C6 and L1 vertebra, L1 (14.7%), T12 (8.2%), and C6 (8.2%) were the most frequently injured segments. In terms of severity, incomplete injury (72.8%) occurred more often than complete injury (27.2%). The American Spinal Injury Association impairment scale of most patients did not convert before and after treatment in the operational group (71.6%) or the conservative group (80.6%). A total of 975 patients (37.2%) from urban and 1,646 patients (62.8%) from rural areas were included; almost all urban residents could rush to get treatment after being injured immediately (<1 h), whereas most rural patients get the treatment needed 4–7 h after injury. The rough annual incidence from 2017 to 2020 is 112.4, 143.4, 152.2, and 132.6 per million people, calculated by the coverage rate of the population of the sampling hospital.

**Conclusion:**

The incidence of TSCI in northwest China is high and on the rise. However, due to pandemic policy reasons, the incidence of urban residents decreased in 2020. The promotion of online work may be an effective primary prevention measure for traumatic diseases. Also, because of the further distance from the good conditional hospital, rural patients need to spend more time there, and the timely treatment of patients from remote areas should be paid attention to.

## Introduction

Traumatic spinal cord injuries (TSCIs) can cause significant morbidity and mortality (1). These injuries are often caused by heavy injuries, traffic accidents, falling accidents, etc. and are centered in the labor age population and elderly population (2, 3). Despite the economic differences between countries or regions, this traumatic disease has caused a large loss of the working population, imposing a serious economic burden on patients and families, which leads to high health expenditure and economic losses (4, 5). Unfortunately, there is currently no effective treatment for patients with TSCI—severe damage to the spinal cord usually means permanent impairment (4, 6, 7). Therefore, attention should be paid to primary prevention. Understanding injury risk factors, incidence, and demographic characteristics can better guide the promotion of preventive measures and the allocation of medical resources (8, 9).

The global incidence of TSCI was 10.5 cases per 100,000 persons, but the incidence of TSCI varies across countries and regions (1). China is a country with rapid industrial development and frequent traffic flow; this brings more injury-causing factors, and the spinal trauma caused by it increases yearly (10). Therefore, it is necessary to update Chinese TSCI incidence data in real time. However, most existing studies have focused on East China, while there has been a lack of extensive multicenter review of TSCI epidemiology in northwest China in recent years (6, 11–14). The level of economic development in northwest China is far behind that in East and mid-China regions, and their characteristics of injury factors should be different, so its epidemiological data cannot fully refer to the data in the East and mid-China regions.

In this study, we aimed to discuss the epidemiological characteristics and risk factors of TSCI in northwest China. Thus, we used the multicenter retrospective data from 2017 to 2020 to expand the coverage area of previous studies and discuss the impact of Corona Virus Disease 2019 (COVID-19) on society (15).

## Materials and methods

### Location and participants

**Figure 1:**
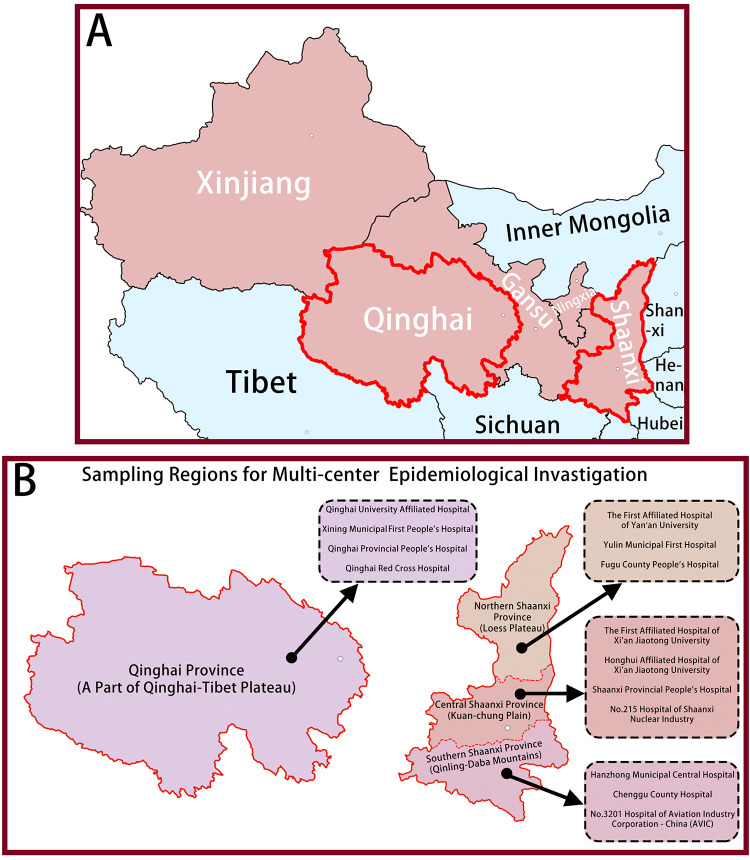
A total of 2621 patients form 14 hospitals in two provinces with TSCI were identified in this study (**A**) Sampling Provinces; (**B**) Sampling Regions for Multi-center Epidemiological Invastigation.

Northwest China comprises five provinces or autonomous regions: Shaanxi, Gansu, Ningxia, Qinghai, and Xinjiang, with a total population of 102.8 million. We chose Shaanxi and Qinghai, two provinces with representative development levels, as shown in Figure 1A. Shaanxi (SN) Province is a traditional industrial and agricultural region with a population of 38.8 million; Qinghai (QH) Province is an animal husbandry region rich in mineral resources, located in the northern part of the Qinghai–Tibet Plateau with a population of 6.1 million. In order to better distribute the research work, Shaanxi is further divided into northern (SN-N), central (SN-C), and southern parts (SN-S) according to climate and physiognomy. We dispatched investigators to the four regions separately and selected three to four hospitals with different administrative levels (provincial, municipal, and county) in each region, a total of 14 hospitals, as shown in Figure 1B. The patients’ information was gathered from the medical records of these 14 hospitals between January 2017 and December 2020.

### Study settings

Eligible patients were screened by the International Classification of Diseases, Version 10 (ICD-10) and its diagnostic code of TSCI. The final diagnosis was based on the patient's diagnosis at discharge/death. Four researchers retrospectively reviewed the medical records of 2,621 patients with TSCI admitted to the 14 hospitals in these two representative provinces between January 1, 2017, and December 31, 2020. The acquired information from patients’ medical records included the patients’ age, gender, occupation, marital status, time of injury, cause of injury, level of injury, severity of the injury, acceptance of surgical treatment, operative mode/approach, damaged segments, preoperative and postoperative scores, rehabilitation therapy, hospital duration, medical costs, and so on.

### Executive organization and ethics statement

This project was jointly undertaken by the China Center for Disease Control (China CDC) and Honghui Hospital of Xi’an Jiaotong University. This study was approved by the ethics committee of Honghui Hospital of Xi’an Jiaotong University. The institutional review boards of the sampled hospitals approved the review process and waived the requirement to obtain patients’ written informed consent.

### Statistical analysis

All numerical data conforming to normal distribution were expressed as the mean ± standard deviation. The analysis of variance and *χ*^2^ tests were used to analyze continuous and categorical data, Wilcoxon rank-sum tests were applied to examine the differences between the non-normally distributed continuous variables, and frequency analysis was used for examining data and calculating percentages. The experimental data were analyzed by SPSS 22.0 (SPSS Inc., Chicago). The figures were made by GraphPad Prism7 (GraphPad Software, CA, United States). *P* < 0.05 were considered as significant difference.

## Results

### General demographic characteristics of patients with TSCI from 2017 to 2020

**Figure 2:**
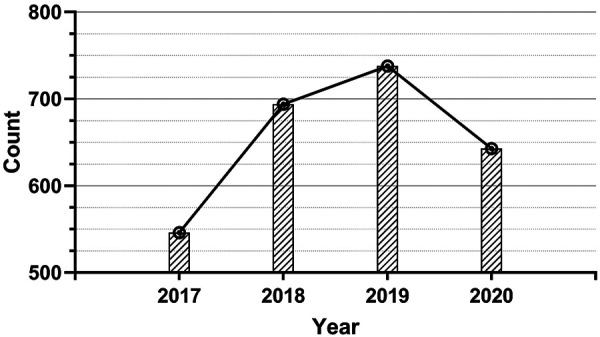
The annual count of TSCI patients during 2017–2020.

A total of 2,621 patients with TSCI from 14 hospitals were identified in this study (Figures 1B, 2). As shown in Table 1, out of these patients, 2,060 were male (78.6%) and 561 were female (21.4%), the male-to-female ratio is close to 3.67:1. The patients’ ages ranged from 6 to 92 years, with an average age of 48 (±14.9) years and a median age of 49 (interquartile range 38) years. Among them, the average age of men and women is 48.0 ± 14.9 and 49.4 ± 14.9 respectively. According to the age distribution, it was found that young adults aged 21–40 years make up half of the TSCI population (49.9%). Regarding the occupation, farmers and herdsmen from rural or pastoral areas account for more than half of the total patients (58.1%), and the patients from urban areas are mainly workers (11.8%), students (5.5%), and retirees (5.6%). The other occupational groups included government officers (1.0%), technicians (1.1%), enterprise managers (1.1%), serviceman (0.2%), and others consisting of freelancers, unemployed individuals, and self-employed individuals who together accounted for 6.9% of the total patients. In addition, 8.7% of patients were unwilling to inform their occupational information for other reasons. Apparently, from 2017 to 2019, there were more patients each year than the previous year, while in 2020, there was a decrease of 12.9% compared with the number of patients in 2019 (Figure 2).

**Table 1:** Demographic and etiologic characteristics of patients with TSCI from 2017 to 2020.

Characters	Years				
	2017	2018	2019	2020	Total
Total	546 (20.8%)	694 (26.5%)	738 (28.1%)	643 (24.5%)	2,621 (100%)
Age (years)					
≤20	18 (3.3%)	21 (3.0%)	34 (4.6%)	40 (6.2%)	113 (4.3%)
21–40	273 (50.0%)	322 (46.4%)	432 (58.5%)	282 (43.8%)	1,309 (49.9%)
41–60	105 (19.2%)	185 (26.7%)	140 (19.0%)	117 (18.3%)	547 (20.9%)
≥61	150 (27.5%)	166 (23.9%)	132 (17.9%)	204 (31.7%)	652 (24.9%)
Gender					
Male	460 (84.2%)	538 (77.5%)	585 (79.3%)	477 (74.2%)	2,060 (78.6%)
Female	86 (15.8%)	156 (22.5%)	153 (20.7%)	166 (25.8%)	561 (21.4%)
Occupation					
Government officer	17 (3.1%)	4 (0.6%)	0 (0.0%)	5 (0.8%)	26 (1.0%)
Technician	1 (0.2%)	9 (1.3%)	18 (2.4%)	0 (0.0%)	28 (1.1%)
Enterprise manager	3 (0.5%)	1 (0.1%)	9 (1.2%)	15 (2.3%)	28 (1.1%)
White-collar worker	22 (4.0%)	4 (0.6%)	1 (0.1%)	1 (0.2%)	28 (1.1%)
Blue-collar worker	51 (9.3%)	73 (10.5%)	98 (13.3%)	59 (9.2%)	281 (10.7%)
Farmer and nomad	310 (56.8%)	421 (60.7%)	413 (56.0%)	378 (58.8%)	1,522 (58.1%)
Student	42 (7.7%)	17 (2.4%)	30 (4.1%)	54 (8.4%)	143 (5.5%)
Serviceman	2 (0.4%)	0 (0.0%)	4 (0.5%)	0 (0.0%)	6 (0.2%)
Freelancer	0 (0.0%)	16 (2.3%)	11 (1.5%)	0 (0.0%)	27 (1.0%)
Self-employed	10 (1.8%)	3 (0.4%)	34 (4.6%)	46 (7.2%)	93 (3.5%)
Unemployed	0 (0.0%)	6 (0.9%)	21 (2.8%)	37 (5.8%)	64 (2.4%)
Retired	43 (7.9%)	32 (4.6%)	31 (4.2%)	40 (6.2%)	146 (5.6%)
Missing^a^	45 (8.2%)	108 (15.6%)	68 (9.2%)	8 (1.2%)	229 (8.7%)
Etiology					
Traffic accidents	120 (22.0%)	158 (22.8%)	237 (32.1%)	111 (17.3%)	626 (23.9%)
Sports and leisure	28 (5.1%)	7 (1.0%)	31 (4.2%)	3 (0.5%)	69 (2.6%)
Slip fall and low fall	159 (29.1%)	185 (26.7%)	161 (21.8%)	222 (34.5%)	727 (27.7%)
High fall	223 (40.8%)	289 (41.6%)	228 (30.9%)	276 (42.9%)	1,016 (38.8%)
Other factors	16 (2.9%)	55 (7.9%)	81 (11.0%)	31 (4.8%)	183 (7.0%)

TSCI, traumatic spinal cord Injury.

^a^ Missing included patients who do not want to disclose information or whose information is not clearly documented.

### Etiology of the patients with TSCI

Analysis of the acquired etiological data showed that high falls were the leading cause of TSCI, indicating 38.8% of total patients (*P* < 0.05), followed by slip falls and low falls (27.7%), traffic accidents (23.9%), and sports (2.6%). Other factors include falling objects, violent fights, and other collisions, accounting for 7.0%. Table 2 shows the etiological composition ratio of TSCI in different age groups, in which there is no apparent difference in etiological composition ratio between ≤20, 21–40, and 40–61 years old groups (*P* > 0.05). However, the proportion of low-energy injury factors (i.e., slip fall or low fall) was higher in patients ≥61 years than in other age groups (*P* < 0.01); nearly half (45.9%) of patients with TSCI over 60 years old are injured by this factor. Table 3 describes the etiological composition of patients of genders. High fall (43.6%) is the most common cause of male patients with TSCI, while slip fall/low fall (37.6%) is the most common cause of female patients with TSCI.

**Table 2:** Etiological composition ratio of TSCI in different age groups.

Age group	Etiology					
	Traffic accidents	Sports	Slip fall/low fall	High fall	Other factors	Total
≤20	30 (26.5%)	16 (14.2%)	33 (29.2%)	27 (23.9%)	7 (6.2%)	113 (100%)
21–40	326 (24.9%)	24 (1.8%)	239 (18.3%)	592 (45.2%)	128 (9.8%)	1,309 (100%)
41–60	138 (25.2%)	15 (2.7%)	156 (28.5%)	203 (37.1%)	35 (6.4%)	547 (100%)
≥61	132 (20.2%)	14 (2.1%)	299 (45.9%)	194 (29.8%)	13 (2.0%)	652 (100%)
Total	626 (23.9%)	69 (2.6%)	727 (27.7%)	1,016 (38.8%)	183 (7.0%)	2,621 (100%)

TSCI, traumatic spinal cord Injury.

**Table 3:** Etiological composition ratio of TSCI in males and females.

Gender	Etiology					
	Traffic accidents	Sports	Slip fall/low fall	High fall	Other factors	Total
Male	439 (21.3%)	53 (2.6%)	516 (25.0%)	898 (43.6%)	154 (7.5%)	2,060 (100%)
Female	187 (33.3%)	16 (2.9%)	211 (37.6%)	118 (21.0%)	29 (5.2%)	561 (100%)
Total	626 (23.9%)	69 (2.6%)	727 (27.7%)	1,016 (38.8%)	183 (7.0%)	2,621 (100%)

TSCI, traumatic spinal cord Injury.

### Injury level

**Figure 3:**
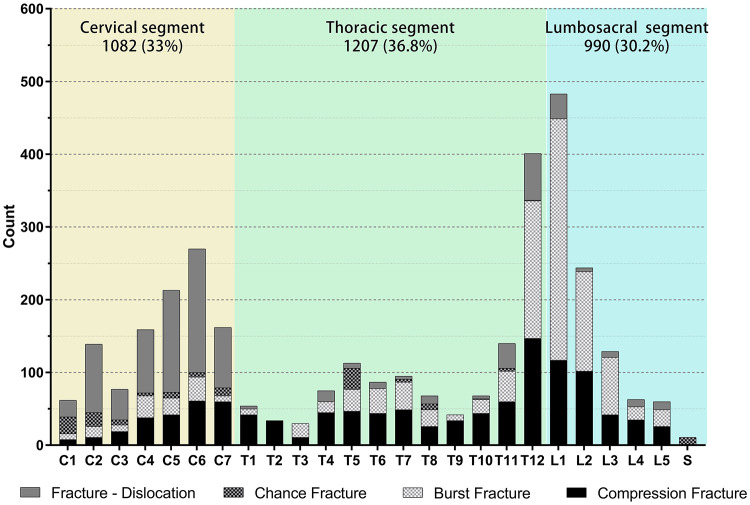
Distribution of fracture level for TSCI patients by the type of fracture (*n* = 2621).

As can be seen from the statistics in Figure 3, TSCI occurred at the cervical, thoracic, lumbosacral levels, and the proportions in these levels were 33.0%, 36.8%, and 30.2%, respectively. We counted the number of cases per injury vertebral segment, and there were 79.0 patients with single-level spinal fractures and 20.1% patients with multilevel (≥2 levels) spinal fractures. Figure 3 shows that the distribution of segmental injury cases showed a “bimodal” pattern; analysis of these data indicates that the two peak injury levels of TSCI were C6 and L1 vertebra. Overall, L1, T12, and C6 were the most frequently injured segments, accounting for 14.7%, 12.2%, and 8.2% of the total cases, respectively. Further, the injury types of each vertebral segment are divided into fracture dislocation, distractive flexion fracture (chance fracture), burst fracture, and compression fracture. Roughly analyzing, the most fracture type of TSCI patients due to cervical injury is fracture dislocation, which accounts for 59.1% of cervical injuries. Compression fractures are more common in TSCI patients due to thoracic injury (exclude T12), which accounts for 48.3% of thoracic injuries; the major fracture type of TSCI patients due to lumbosacral injury (include T12) is burst fracture, which accounts for 55.9% of thoracic injuries.

### Severity of TSCI

**Figure 4:**
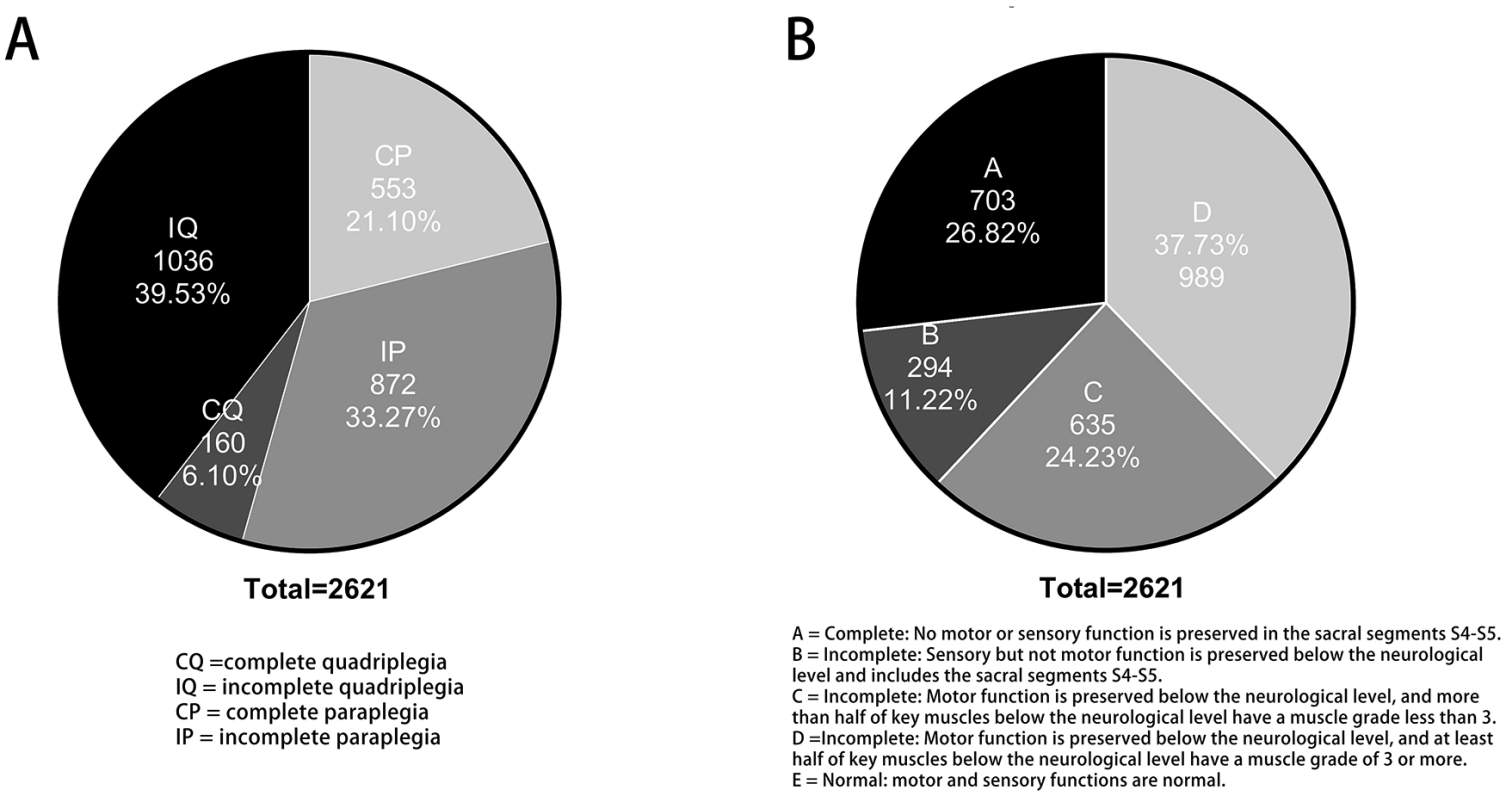
The severity of patients with TSCI: (**A**) The degree of injury; (**B**) Their spinal function after injury evaluated by ASIA impairment scale.

The severity of patients with TSCI was divided into complete quadriplegia (CQ), incomplete quadriplegia (IQ), complete paraplegia (CP), and incomplete paraplegia (IP) according to the degree of injury, as shown in Figure 4A. Most patients with TSCI present with IQ, accounting for 39.5%. The next is IP, accounting for 33.3%. Patients who suffered from complete injury included CQ and CP, accounting for 21.1% and 6.1%, respectively. Admission assessment results using the American Spinal Injury Association (ASIA) impairment scale system are shown in Figure 4B. From the pie chart of patients with TSCI patients, 26.8% of patients suffered from complete motor and sensory dysfunction (ASIA A), 11.2% suffered from complete motor dysfunction with some part of the sensory function retained (ASIA B), 24.2% had inefficient motor functions (myodynamia of most key muscles <3, ASIA C), and 37.7% had useful motor functions remain (myodynamia of key muscles >3, ASIA D).

### The period between injury and admission

**Figure 5:**
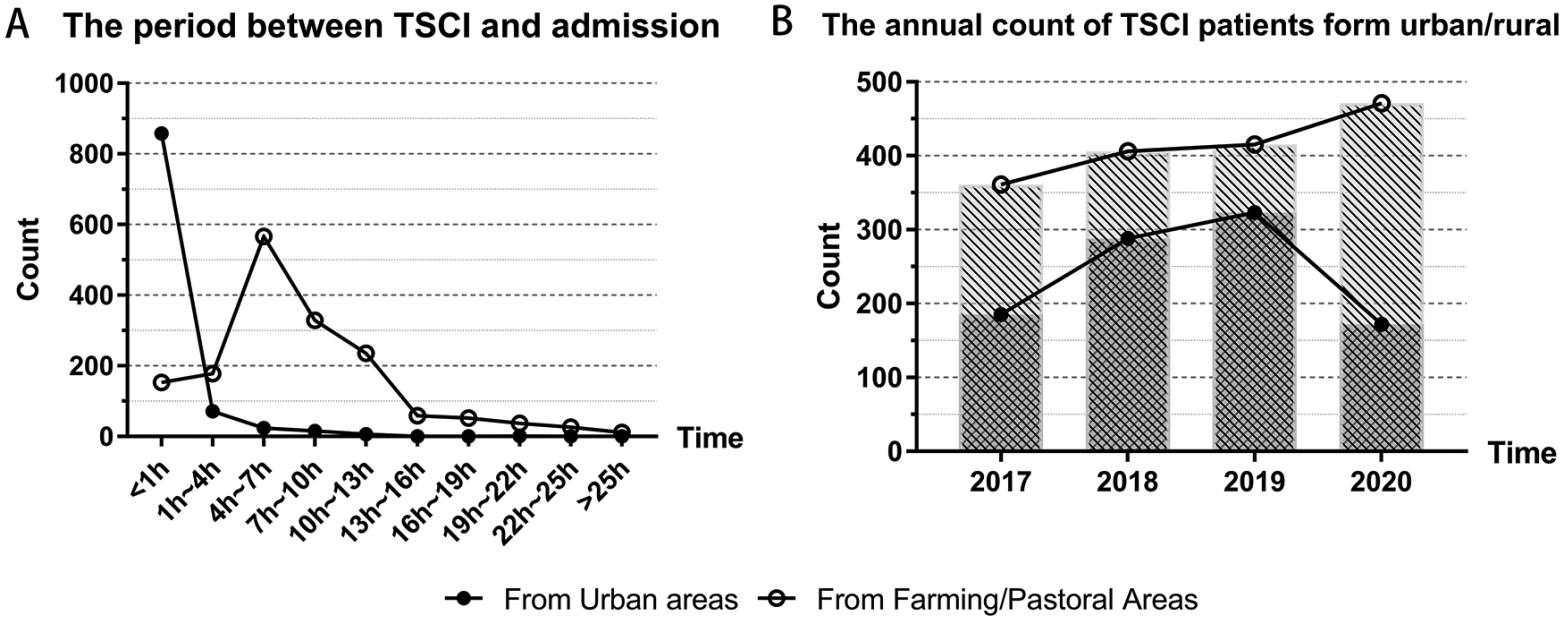
Urban and rural differences in patient treatment: (**A**) The patient's waiting time from injury to admission; (**B**) The annual count of TSCI patients form urban and rural.

We recorded the patient's waiting time from injury to admission. According to the data characteristics, we counted the 975 patients from urban and 1,646 patients from rural areas with TSCI separately in Figure 5. It was observed that most urban residents (88% of 1,646 patients) were able to rush to hospital for medical treatment within 1 h of injury. When most patients (55.4% of 1,646) with TSCI from rural areas arrived at regional hospitals which are qualified for treatment, 4–7 h had passed since the time of injury.

### The annual count of TSCI patients form urban and rural

We divided the annual patient count into two groups: those from urban and those from rural areas. From 2017 to 2020, the annual number of urban patients was 185, 288, 323, and 172, which showed an increasing trend in the first three years, and decreased apparently in 2020 compared with 2019. From 2017 to 2020, the number of rural patients was 361, 406, 415, and 471, and the number of cases had been rising for four consecutive years.

### Treatment of TSCI and status on discharge

In terms of the treatment that TSCI patients received, 2,002 cases received operative treatment and 619 cases received conservative treatment. We regard the outcomes of the patients treated with operative or conservative treatment as a whole. We can figure out the change in patients’ condition after receiving one of the treatments from the data in Table 4. Regardless of the treatment, about three-quarters of patients had no change in their ASIA impairment scales before and after treatment; among them, the patients who received surgical treatment accounted for 71.6% and the patients who received conservative treatment accounted for 80%. If the ASIA scale was improved on admission but still at an incomplete level (B/C/D), it would be regarded as improvement, with an improvement rate of 22.4% for those treated surgically 11.6% for those treated conservatively, about half of the former. Patients with the ASIA scale of Grade E at discharge were considered cured; the curing rate of both methods was unsatisfactory, being 4.8% for operative treatment and 7.1% for conservative treatment. In addition, there were a few cases of deterioration or death.

**Table 4:** Treatment of TSCI and functional changes in discharge and admission.

Treatment of TSCI	Status on discharge					
	Cure^a^	Improvement^b^	Unchanged^c^	Deterioration^d^	Death	Total
Operative treatment	97 (4.8%)	448 (22.4%)	1,433 (71.6%)	11 (0.5%)	13 (0.6%)	2,002 (100.0%)
Conservative treatment	44 (7.1%)	72 (11.6%)	499 (80.6%)	0 (0.0%)	4 (0.6%)	619 (100.0%)
Total	141 (5.4%)	520 (19.8%)	1,932 (73.7%)	11 (0.4%)	17 (0.6%)	2,621 (100.0%)

^a^ At discharge, the ASIA scale reached grade E.

^b^ ASIA scale was improved than that on admission but still at an incomplete level (B/C/D).

^c^ ASIA scales on admission and discharge were the same.

^d^ ASIA scale was worse than that on admission.

TSCI, traumatic spinal cord Injury; ASIA, American Spinal Injury Association.

### Estimation of TSCI incidence in northwest China

We can only infer the whole from the approximate relationship between the number of beds in our sampling hospital and the regional population covered by them. By referring to the China Statistical Yearbook (https://www.yearbookchina.com/) from 2017 to 2020, we combined the bed data of the five provinces in northwest China and concluded that the average annual bed number in northwest China during the 4 years was 6.194 beds per thousand people. The total number of beds in the 14 sampled hospitals was about 30,000, so it is estimated that the hospitals could cover a population of 30,000/6.194 × 10^−3 ^= 4,843,397. The 4-year incidence rate of TSCI can be estimated as the ratio of TSCI patients admitted in the hospital to the total population covered by the hospital. These hospitals treated 2,621 patients with TSCI in the 4 years from 2017 to 2020. If we use these samples to estimate the population, the 4-year incidence rate of TSCI (per million people) was calculated to be 541.15 cases in the population covered by these hospitals. According to the survey data (Figure 2), the annual incidence rates from 2017 to 2020 were 112.4, 143.4, 152.2, and 132.6 per million people, respectively.

## Discussion

Northwest China is an economically backward region in China with low coverage of health insurance and educational level, but it is still developing rapidly. Compared to eastern areas of China, such as Shanghai, Beijing, and Guangdong, northwest China has several unique characteristics. For example, the proportion of TSCI patients over 60 years in northwest China (24.9%) was higher than that in eastern regions. In addition, the proportion of farmers/nomads (58.1%) in the patients was apparently higher than that in the east (11, 16, 17). Many people are engaged in traditional manual labor or industrial production. Traffic factors and falls are both important injury factors for TSCI in the labor force (industry, agriculture, and husbandry). In the past, we have conducted single-center studies (15). However, despite its underdevelopment with low population density, the total area of five provinces in northwest China is nearly 3.08 million square kilometers, which is a vast area that covers about one-third of the land area of China. To get timely treatment, local patients usually choose a nearby medical center. Therefore, multicenter studies in multiple regions can better represent the whole region than single-center research. We collected the data of patients with TSCI admitted to 14 hospitals located in QH, SN-N, SN-C, and SN-S to understand the changes in the incidence of spinal cord injury patients before and after the emergence of COVID-19 (2017–2020). It can help optimize the allocation of medical resources and provide timely healthcare to the population of more areas.

Annual count results demonstrated an increasing trend in TSCI patients from 2017 to 2019. This trend is consistent with previous research findings (6, 15, 18). Unusually, the patient count in 2020 was apparently lower than that in the previous year; there were 12.8% fewer patients in 2020 than in 2019. We speculate that this is due to the shutdown and some “work-at-home” proposals implemented by the Chinese Government in the first two quarters of 2020 to prevent the COVID-19 epidemic. Recent studies on other traumatic diseases have reached similar conclusions (19–21). In addition, from the perspective of patients from urban or rural areas, the impact of these policies on the urban population is far greater than that on the rural population; the number of patients in the urban population in 2020 is only 53.3% of the last year, while the number of patients in rural areas in 2020 is still increasing compared with that in the previous year. We consider that this is related to the feasibility of shutdown policies and “work-at-home” proposals. In cities, where there are more office workers and students, it is easier to work or study online, which largely shields them from exposure to many outdoor injury factors, while agricultural or livestock production is hard to move online. Another indication is that the proportion of patients aged over 60 years in 2020 shows an increase. Due to degenerative bone changes and hypofunction of sensory and motor, nontraffic and low-energy injury factors are more likely to cause TSCI in elderly people than young and middle-aged people (22, 23). Combined with the epidemic policy mentioned earlier, this evidence is consistent with our conclusions. Although the COVID-19 pandemic brings immeasurable economic losses and damages to human health, it also facilitates the development of online work and learning, which makes it possible for many jobs to be performed online from home for a long time. In our conclusion, promoting online office work and learning will make a lot of sense to reduce the incidence of traumatic diseases among people at labor age, especially for urban residents.

In our investigation, the male-to-female ratio in patients with TSCI was 3.67:1, which is similar to the rate reported in other regions (6, 14, 24). This indicates that most of the patients with TSCI are male. This can be attributed to the fact that most workers in dangerous, physically demanding jobs are male and a greater proportion of drivers are male.

Most of the injuries were in the 21–40 age group (49.9%, 1,309 cases), followed by the ≥61 age group (24.9%, 652 cases) and 41–60 age group (20.9%, 547 cases). There are two age groups with high incidence. Such a “bimodal” trend seems to be different from the conclusions of previous studies. Most of the previous literature works described a “unimodal” trend with the highest age group around 40 ± 10 years old (6, 17, 25). This may be due to the difference in group spacing and the increased incidence of the elderly under the 2020 epidemic policy. The mean age of patients in our data was 48 (±14.9) years, which was higher than the domestic average age and global average age suggested by previous literature works (10, 26). We estimate that this result may be influenced by the aging of society and the policy of delaying retirement time.

Our study also revealed the etiology of the patients with TSCI in northwest China, including, traffic (23.9%), sports (2.6%), low falls (27.7%), high falls (38.8%), and other factors (7.0%). Groups were observed according to age first; we found that high-energy injury factors, such as high falls, were the most common cause of TSCI in 21–40 and 41–60 age groups, accounting for 45.2% and 37.1%, respectively, and in people over 60 years, low-energy factors, such as low falls or slips, accounted for 45.9% of the total cases. This is consistent with the conclusion of the study in Guangdong Province (6). Then, groups were observed according to gender, and the result indicated that males were more likely to have been impacted by high-energy factors causing TSCI.

Integrating the above etiological results, the age of high incidence of TSCI is still dominated by the labor age, especially in males. So, the labor security agencies of governments and the employers of workers should also strengthen labor safety measures and enhance safety education for employees (13). In addition, specialization and mechanization of agricultural and animal husbandry workers should be promoted so that dangerous and strenuous manual labor can be as far as possible from machines.

Spinal cord injury is usually associated with spinal trauma injury, and the levels of injury are corresponding. We counted the segments of the patient's vertebra and the types of fractures; it is the same as the previous literature; the proportions of injuries in cervical, thoracic, and lumbar vertebrae were similar, and the high incidence of spinal fractures caused by TSCI in each part was C5–C6, T11–T12, and L1–L2. The distribution of injury segments showed a “bimodal” distribution with C6 and L1 as the centers, with 1–2 adjacent segments (25, 27). Overall, the T11–L3 segment had the highest proportion of injuries, totaling 1,397 cases, accounting for 42.6% of all injured patients. The main fractural types of cervical, thoracic, and lumbar vertebrae are also different, which is related to their anatomical structures and mechanical characteristics.

From the severity of the injury and the outcome of treatment, our results revealed a less optimistic condition after treatment. As a result of consensus, incomplete injury (72.8%) occurred more often than complete injury (27.2%). Given the large number of patients with incomplete TSCI, this issue should be the focus of basic research related to neural regeneration, such as “how to promote the compensation of surviving neurons in the injured area” or “how to achieve differentiation of uninjured stem cells into neurons in the incomplete injured segment” (28). The ASIA impairment scale of most patients did not change before and after treatment in both the operational group (71.6%) and the conservative group (80.6%). This illustrates the importance of prevention in improving tertiary prevention measures and rehabilitation techniques as an essential guarantee of improved treatment outcomes. Moreover, the patients who were treated operationally had a higher improvement rate (22.4%) and deterioration rate (0.5%) than the patients who were treated conservatively (improvement rate was 11.6%, deterioration rate was 0.0%). Therefore, improving surgical methods to reduce postoperative complications is also a strategy to improve the efficacy of TSCI.

Our study also focused on urban–rural differences that had not previously been noticed by investigators. The annual difference in the number of cases was mentioned earlier, and we also compared the length of time between the onset and the treatment of patients in urban and rural areas. From our results, we can see that almost all urban residents can rush to the hospital for emergency treatment after getting injured immediately (<1 h), whereas by the time most patients from rural areas arrive at the hospital for treatment, it has been 4–7 h since they were injured. This reflects the delay in treating patients caused by poor transport conditions in rural areas. In fact, some hospitals in developed areas are already using helicopter emergency medical services (HEMS) to save treatment time for patients in remote areas (29). This kind of traffic measure is not restricted by the topography and is of great value for patients with various acute diseases in rural areas such as the Loess Plateau and the Qinghai–Tibet Plateau in northwest China. The problem is the high cost of HEMS, which also requires better allocation of resources and funding by the public health system.

Finally, we estimated the incidence of TSCI in northwest China based on the hospital coverage population from 2017 to 2020; the incidence rate ranged from 112.2 to 152.4 cases per million people, which is more than 23.7 per million people in Tianjin and 60.6 per million people in Beijing (16, 30). The incidence of TSCI is difficult to calculate due to the unpredictability of the occurrence of trauma. Sampling methods, inclusion criteria, regional demographic differences, and other factors will affect the results. Our estimated results only provide a reference for the incidence of TSCI in northwest China, and more scientific design and observational studies are needed to obtain its incidence accurately.

However, as a retrospective study, there are irreparable misrecords or incomplete information in the data we obtained, which may lead to a deviation between our results and the actual situation. Furthermore, we ignore the data on treatment cost and Medicare coverage, which can well reflect the economic pressure of patients and the development level of the region. Therefore, there is insufficient evidence in some descriptions of the severity of TSCI in northwest China.

## Conclusions

In general, the incidence of TSCI in northwest China is high and on the rise. Due to the implementation of COVID-19 prevention and control measures, the incidence of TSCI among urban residents has decreased to a certain extent. Therefore, we suggest that promoting online office and learning is the effective primary prevention measure for traumatic diseases. In addition, due to the differences between urban and rural areas, rural patients need to spend more time getting to a good conditional hospital for treatment, and the problem of emergency transfer service still needs to be addressed.

## Data Availability

The raw data supporting the conclusions of this article will be made available by the authors, without undue reservation.
